# Empirical Analysis of Early Childhood Enlightenment Education Using Neural Network

**DOI:** 10.1155/2022/3601941

**Published:** 2022-08-29

**Authors:** Jingyi Cheng, Jianjun Cheng

**Affiliations:** ^1^School of Education, Taylor's University, Petaling Jaya, Selangor 47500, Malaysia; ^2^Computer School, Hubei University of Arts and Science, Xiangyang, Hubei 441053, China

## Abstract

This exploration aims to study the value orientation and essence of early childhood enlightenment education based on the deep neural network (DNN). Based on the acquisition and feature learning of cross-media education big data, the DNN correlation learning of cross-media education big data, and the intelligent search of cross-media education big data based on the DNN, the intelligent search system of cross-media children's enlightenment education big data based on DNN is designed and implemented. The system includes three functional modules: the feature learning module of cross-media infant enlightenment education big data, the deep semantic correlation learning module of cross-media childhood enlightenment education big data, and the intelligent search module of cross-media childhood enlightenment education big data based on DNN. This exploration realizes the acquisition and feature learning of big data of cross-media early childhood enlightenment education, DNN learning of cross-media education big data of early childhood enlightenment, and intelligent computing of cross-media education big data based on DNN. The experimental results show that the proposed system's mean average precision (MAP) performance is improved by 15.6% on the public dataset of early childhood enlightenment education published by the Ministry of Education. Moreover, the system has also significantly improved the MAP performance of the constructed dataset in the field of early childhood enlightenment education; that is, the MAP performance has been improved by 20.6% on the dataset of Taylor's University in Malaysia (NUS-WIDE). This exploration has certain theoretical significance and empirical value for early childhood enlightenment education research.

## 1. Introduction

“Enlightenment education” is something that every parent attaches great importance to. Many parents have prepared multiple enlightenment toys before their children are born, such as various building blocks, mental training picture toys, and thinking training cards [1]. However, in reality, sometimes, parents do not know where to start enlightenment education for their children. When will children become enlightened? It is a problem that parents attach great importance to [2]. In the past, an educational scholar used radioactive sugar experiments to observe the brain cell activities of children of different ages. The results show that the brain activity of 2-year-old children is exactly the same as that of adults. The brain activity of 3- to 12-year-old children is very active, and the brain activity is twice that of adults. However, the level of brain activity in children aged 13-14 has returned to the same level as that in adults [3–5]. Meanwhile, under the “enlightenment” curriculum concept, teachers and children follow China's excellent traditional etiquette culture. From a “child's perspective,” Kindergartens always adhere to preschool education's comprehensive enlightenment education method, which is generally divided into five fields: health, language, society, science, and art. The contents of each field permeate each other and promote the development of children's emotions, attitude, ability, knowledge, and skills from different angles [6, 7].

In ancient Eastern and Western societies, education was closely related to religion. It was believed that the purpose of education was to reflect the will of God. Due to the relatively low level of scientific development, the cognition of human beings and the origin of education were also limited by history [8]. The theory of biological origin holds that educational activities exist in human social life and the animal kingdom. This theory denies human sociality and simply attributes the complex social activity of education to animal instinct [9]. According to the theory of psychological origin, in primitive society, children only learn life knowledge and skills by observing adults and trying successful methods. This theory ignores society's key role and attributes children's education to imitation behavior in the unconscious state [10]. Qi et al. showed that parents or teachers more pursued educational tools and practicality in the current early childhood education, paid more attention to the immediate “leading” effect, and despised or even ignored the enrichment and long-term development of children's ideas [11]. Kim held that this utilitarian educational tendency violated the essence of early childhood education and harmed the whole cause of early childhood education [12]. Li et al. studied the psychological changes of early childhood education in China and proposed the implementation of early childhood education in line with children's physical and mental development [13]. Buronova and Abdunazarova proposed that education was obtained in life, and education was the process of encouraging children to experience everything and gain direct experience through subject activities [14]. The above research theories and the research of children's psychology provide educational significance and reference for children's enlightenment education. However, there is no research on early childhood education's value orientation and essence.

First, this exploration discusses the background of early childhood enlightenment education and education-related theories, providing background discussion for this exploration. Next, it describes the process of the deep neural network (DNN) algorithm, the feature learning of cross-media education big data, and the process of network association learning to provide technical guidance for this exploration. The main research innovation lies in the establishment of a cross-media big data intelligent search system for early childhood education based on DNN, which is an outstanding contribution of the research to early childhood education. Finally, on different datasets and different neural network algorithms, the intelligent search results of cross-media education big data in early childhood education are analyzed.

## 2. Method

### 2.1. Neural Network Algorithm and Artificial Neuron Model

**Figure 1:**
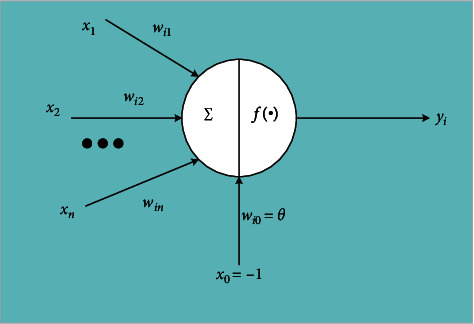
Artificial neuron model.

The neural network is a computing model composed of massive nodes and their connections. Each node represents a specific output function called the activation function. In essence, the network itself is usually an approximation of some algorithm or function or an expression of logical strategy. An artificial neuron is the basic element of a neural network.  Figure 1 shows the artificial neuron model.

In Figure 1, *x*_1_ ~ *x*_*n*_ are input signals from other neurons, *w*_*ij*_ represents the connection weight from neuron *j* to neuron I, and *θ* represents the threshold, also known as deviation [15,16]. The bias effect is to increase or decrease the network input of the activation function according to whether it is positive or negative. Equations (1) and (2) are the relationship between the output and input of a neuron:

(1) $$\begin{array}{c} {\text{net}}_{j}=\sum_{j=1}^{n}w_{ij}x_{j}-\theta , \end{array}$$

(2) $$\begin{array}{c} y_{i}=f\left({\text{net}}_{i}\right). \end{array}$$

*y*_*i*_ represents the output of neuron *i*, function *f* is the activation function, and net is the net activation. If the threshold is regarded as the weight *w*_*i*0_ of an input *x*_0_ of neuron *i*, equation (3) is the output of neuron *i*:

(3) $$\begin{array}{c} y_{i}=f\left({\text{net}}_{i}\right)=f\left(\sum_{j=0}^{n}w_{ij}x_{j}\right). \end{array}$$

If the neuron's net >0, the neuron is activated; if the neuron's net <0, the neuron is inhibited. The neural network is a network interconnected by massive neurons. According to the interconnection mode of neurons in the network, the common neural network structure is shown in Table 1.

**Figure 2:**
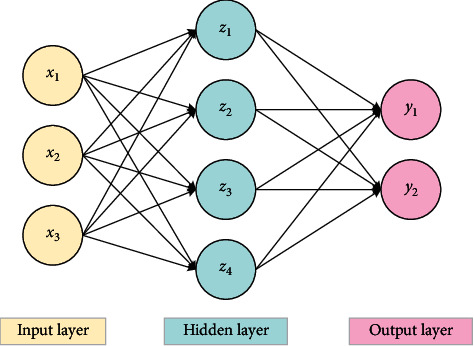
The structure of DNN.

DNN can be understood as a neural network with many hidden layers, also known as a deep feedforward network (DFN).  Figure 2 displays the structure of DNN.

Figure 2 shows that multiple neurons are intertwined to form a network structure called DNN. DNN usually consists of input, hidden, and output layers [17,18]. The number of neurons in the input layer is equal to the number of characteristic attributes of the input sample. The number of hidden layers can be customized (generally more than 3 layers), and the hidden layer data are not visible. Equation (4) represents its linear relationship:

(4) $$\begin{array}{c} z=\sum_{i=1}^{m}w_{i}x_{i}+b. \end{array}$$

The output layer is the output part of the model. The number of neurons is determined by the number of class markers to be classified. Therefore, the number of output elements is not necessarily the same as the number of inputs. Under the three input data, the output value of the model is 2. Layers are fully connected, and any neuron in layer *i* must be connected with any neuron in layer *i* + 1. According to the number of hidden layers, the parameters of DNN will increase, and certain rules are required for the definition of linear relationship coefficient *w* and offset *b*. The linear coefficient from the fourth neuron in the second layer to the second neuron in the third layer is defined as *w*_24_^3^. The superscript 3 represents the number of layers of the linear relationship coefficient *w*, while the subscript corresponds to the output third layer index 2 and the input second layer index 4. *w*_24_^3^ transpose is required during matrix operation. If the output index is put in front, the linear operation does not need to transpose. Moreover, it should be noted that the input layer has no *w* parameter and bias parameter *b*. The offset corresponding to the third neuron in the second layer is defined as *b*_3_^2^. Among them, superscript 2 is the number of the layer where it is located, and subscript 3 represents the index of neurons where the bias is located.

The forward propagation algorithm of DNN is a series of linear operations and activation operations starting from the input layer, using the input vector, several weight coefficient matrices, and bias vectors. It uses the previous layer's output to calculate the output of the next layer. The backward calculation is conducted layer by layer until the output layer obtains the output result. If the number of neurons in layer *l* − 1 is *m*, the output of the *j*-th neuron in layer *l* is recorded as *a*_*j*_^*l*^. Its calculation expression reads

(5) $$\begin{array}{c} a_{j}^{l}=\sigma \left(z_{j}^{l}\right)=\sigma \left(\sum_{k=1}^{m}w_{jk}^{l}a_{k}^{l-1}+b_{j}^{l}\right). \end{array}$$

In (5), if *l*=2, the corresponding *a*_*k*_^*l*−1^ is the input layer *x*_*k*_, and *l* is the total number of layers. The forward propagation is adopted to calculate the output of the training sample, and the loss function is used to measure the loss between the calculated output of the training sample and the label of the real training sample. DNN's backpropagation (BP) algorithm iteratively optimizes the loss function with the gradient descent method to find the minimum value. It finds the appropriate linear coefficient matrix *W* and offset vector *b* corresponding to the hidden layer and output layer and makes the calculated output of all training sample inputs equal to or close to the sample label as much as possible. The mean square error is used to measure the loss. For each sample, the expression of expected minimization reads

(6) $$\begin{array}{c} J\left(W,b,x,y\right)=\frac{1}{2}a^{L}-y_{2}^{2}. \end{array}$$

In (6), *a*^*L*^ and *y* are output vectors, and *S*_2_ is the *L*2 norm of S. Next, the gradient descent method is used to solve the *W* and *b* of each layer pair. Equations (7) to (9) are the calculation expressions of *W* and *b* of layer L:

(7) $$\begin{array}{c} \frac{\partial J\left(W,b,x,y\right)}{\partial W^{l}}=\frac{\partial J\left(W,b,x,y\right)}{\partial z^{l}}\frac{\partial z^{l}}{\partial W^{l}}=\delta^{l}{\left(a^{l-1}\right)}^{T}, \end{array}$$

(8) $$\begin{array}{c} \frac{\partial J\left(W,b,x,y\right)}{\partial b^{l}}=\frac{\partial J\left(W,b,x,y\right)}{\partial z^{l}}\frac{\partial z^{l}}{\partial b^{l}}=\delta^{l}, \end{array}$$

(9) $$\begin{array}{c} \delta^{l}=\delta^{l+1}\frac{\partial z^{l+1}}{\partial z^{l}}=\left(W^{l+1}\right)\delta^{l+1}⊙\sigma^{\prime }\left(z^{l}\right). \end{array}$$

It can be found that there is a common part (*∂J*(*W*, *b*, *x*, *y*))/*∂z*^*l*^ in the equation for solving *W* and *b*, which is recorded as *δ*^*L*^. *δ*^*L*^ represents the gradient of the inactive output *z*^*l*^ of layer *l*. ⊙ stands for Hadamard product. In the training process, if the change values of all *W* and *b* are less than the stop iteration threshold *ϵ*, it is necessary to calculate the linear relationship coefficient matrix and offset vector between each hidden layer and the output layer again.

In the BP algorithm, there is a lot of continuous multiplication due to the chain rule of matrix derivation. If the number of successive multiplications of each layer is less than 1, the gradient will decrease with progress, resulting in the disappearance of the gradient. However, if the number of successive multiplications of each layer is greater than 1, the gradient is multiplied forward, and the gradient increases, resulting in gradient explosion. Gradient explosion can generally be solved by adjusting the initialization parameters in the DNN model. When the gradient disappears, the leaky rectified linear unit (LReLU) activation function can be used to solve it to a certain extent. The calculation expression reads

(10) $$\begin{array}{c} f\left(z\right)=\left\{\begin{array}{c} z,z>0,\\ az,z\leq 0. \end{array}\right. \end{array}$$

The difference between LReLU and rectified linear unit (ReLU) is that when *z* ≤ 0, its value is not 0, but a linear function with slope *a*. Generally, *a* is a small positive number, which realizes unilateral suppression, and retains some negative gradient information without complete loss. The parameter value of *a* needs repeated training for many times to be determined.

### 2.2. Feature Learning and Network Correlation Learning of Cross-Media Education Big Data

**Figure 3:**
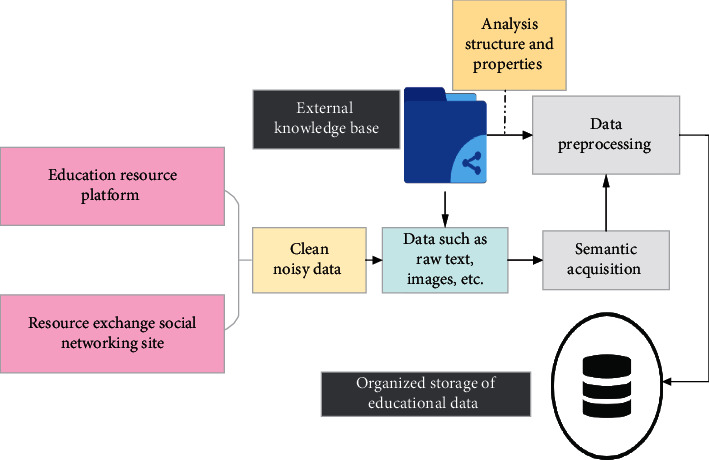
Acquisition process of cross-media education big data.

With the penetration of large-scale information network technology in the field of education, unprecedented big data resources are gradually formed and accumulated, including text, image, video, and other cross-media data. These huge data contain rich and valuable information. However, massive heterogeneous and diversified cross-media big data are distributed on various social networks and other multisource Internet platforms. If users want to find the effective information they need in the massive data, the traditional information search technology has been difficult to meet users' growing personalized and accurate information acquisition needs. Thereby, it is necessary to improve the accuracy of resource search, fully use the big data-driven artificial intelligence technology, and carry out in-depth research on the intelligent and accurate search of cross-media big data. It has important theoretical significance and wide application value [19, 20].  Figure 3 shows the acquisition process of cross-media education big data.

**Figure 4:**
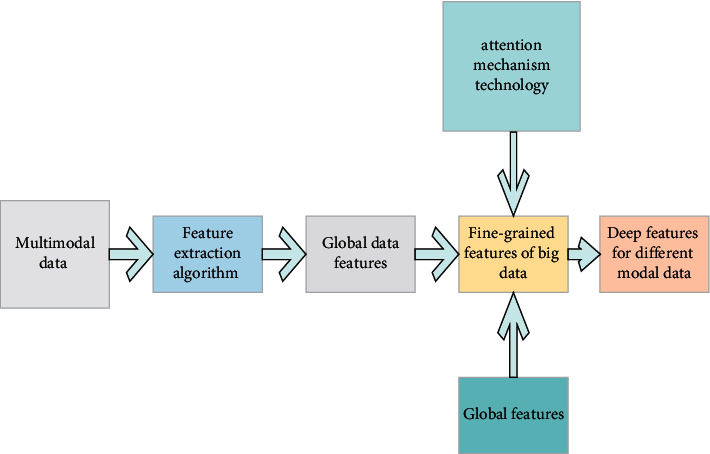
Feature learning process of cross-media education big data.

In Figure 3, first, the image, text, and other data in early childhood education big data are extracted. The noise of data is the inaccurate data describing the scene of early childhood education. It will increase the amount of data and calculation and increase the computer's internal storage and calculation error in the process of model training. Here, the distributed binning is used to process the data noise. First, according to the subinterval divided by the educational attribute value (whether it belongs to early childhood enlightenment education), if an attribute value is within a certain subinterval, it is said to put the attribute value into the “box” represented by this subinterval. The data to be processed (a column of attribute values) are put into some boxes according to certain rules. The data in each box are investigated. The deep learning (DL) training method is used to process the data in each box. Moreover, after the acquisition of big data of early childhood enlightenment education, Figure 4 displays the characteristic learning process of big data of cross-media education.

**Figure 5:**
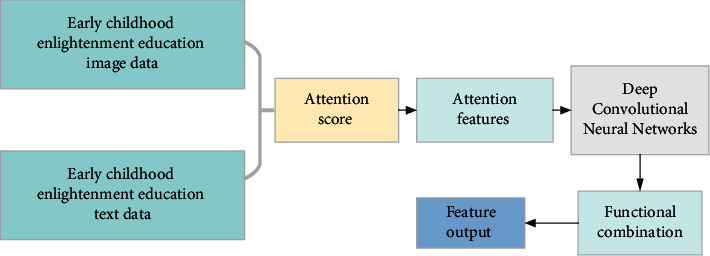
Attention mechanism of visual semantic union.

In Figure 4, for the text data of early childhood enlightenment education, DNN is used to extract the text features. For the image data of children's enlightenment education, deep convolution operation and digital processing of speech signals are adopted. The convolutional neural network (CNN) allows multiscale features in different convolution layers to be used as input and extracts output feature maps of the same scale. The attention mechanism realizes the feature alignment of multimodal data of early childhood enlightenment education by weighting the output of the encoder. The attention mechanism of visual semantic union is used to mine the fine-grained association of multimodal data of early childhood enlightenment education.  Figure 5 shows the attention mechanism of the visual semantic union.

In the process of intelligent and accurate search, semantic reasoning and matching can be carried out according to the situation and purpose of early childhood enlightenment education. It can establish the semantic association between early childhood enlightenment education knowledge and change the search from web search to search with knowledge as granularity to get smarter and more comprehensive recommendations and search results. Besides, the cross-media big data generated by the early childhood enlightenment education platform and related education scene information show strong semantic relevance in semantics. Making full use of this semantic association can mine various network behavior characteristics of early childhood enlightenment education from different dimensions. It helps to establish a comprehensive cross-media big data knowledge association of early childhood enlightenment education and thus plays a good role in promoting the accurate search of cross-media big data [21].

**Figure 6:**
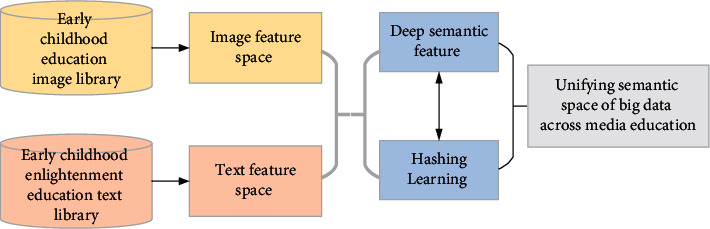
DNN correlation learning process of cross-media education big data.

The characteristics of different forms of educational data, such as text and image, are heterogeneous, and there is a large semantic gap. Therefore, it is impossible to match and search cross-media resources directly in the search process of cross-media education data. Data from different media often have high semantic relevance. Cross-media semantic correlation learning can map the heterogeneous feature space of different media to the unified semantic feature space and establish the common semantic space of different media data. On this basis, cross-media education data search is realized through similarity matching.  Figure 6 shows the process of cross-media learning.

Due to the limited research energy, different algorithms can be used to assist the hash model in hash learning. The algorithms that can be used are similarity-sensitive hashing across modalities (SSHAM), semantic relevance maximization methods (SRMM), unsupervised generative adversarial cross-modal hash algorithms (UGACMHA), self-supervised adversarial hash algorithms (SSAHA), and adversarial guided asymmetric hash algorithms (AGAHA).

### 2.3. Design of Intelligent Search System for Big Data of Cross-Media Early Childhood Enlightenment Education

**Figure 7:**
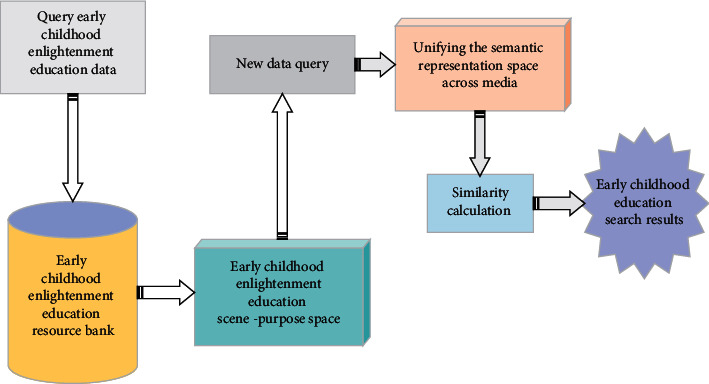
Intelligent search system for big data of cross-media early childhood enlightenment education.

Based on the joint embedding features of the trained visual semantics, the salient region detection network of the image is combined with the attention mechanism to learn the association between the salient regions and the joint embedding layer with emotional semantics in the attention mechanism. The classifier based on these regions performs well in image emotion prediction. As an unsupervised learning method in DL, it has achieved good results in the field of natural language processing. The basic idea of the automatic encoder is to transform the original high-dimensional features into low-dimensional vectors, learn the potential features in the original data, eliminate the redundant parts in the high-dimensional features, and obtain the refined expression of the original data. Based on the above DNN intelligent search of cross-media education big data, a DNN-based cross-media big data intelligent search system for early childhood education is designed and implemented.  Figure 7 shows the big data intelligent search system for cross-media early childhood education.

According to the above intelligent search system for big data of cross-media early childhood education, data query and early childhood education resource database are connected through educational space scenes. The similarity is calculated for the unified cross-media semantic representation space to finally get early childhood education search results. The similarity between the unified semantic representation of early childhood education data query and the unified semantic representation of early childhood education resources is calculated [22]. The results show that the effectiveness of the system flow has been verified. The following equations are the calculation expressions:

(11) $$\begin{array}{c} s\left(Q,R\right)=\cos\;\theta =\frac{Q\cdot R}{QR}, \end{array}$$

(12) $$\begin{array}{c} \text{Sim}\left(EQ,Q\right)=\frac{\sum_{i=1}^{n}\text{Sim}\left(ec_{i},c\right)}{n}, \end{array}$$

(13) $$\begin{array}{c} s=\frac{s\left(Q,R\right)+\sum_{i=1}^{n}\text{Sim}\left(EQ_{i},Q\right)\cdot s\left(EQ_{i},R\right)}{\sqrt{1+\sum_{i=1}^{n}\text{Sim}{\left(EQ_{i},Q\right)}^{2}}}. \end{array}$$

*s*(*Q*, *R*) is the similarity value between the query *Q* of early childhood enlightenment education and the search result *R* of early childhood enlightenment education. *EQ* in *Sim*(*EQ*, *Q*) is the expanded query vector in the search process, *ec*_*i*_ is the query keyword, *c* is the expanded concept of early childhood enlightenment education, *n* is the number of returned results in the search process, and *s* is the similarity score.

### 2.4. Evaluation Method of an Intelligent Search System for Big Data of Cross-Media Early Childhood Enlightenment Education

The selected system evaluation index can be used according to the DL target detection evaluation index. The dataset of model training is the MNIST dataset. This training dataset contains 400 groups of data that have been specially labeled. The learning rate of the model is 0.001, the convolution kernel size is 40, and the batch processing capacity is 30. It is assumed that there are two kinds of original samples, which are actually positive samples and negative samples. True positives (TP) represent true positive samples, true negatives (TN) represent true negative samples, false positives (FP) represent false positive samples, and false negatives (FN) represent false negative samples. The precision of the search task during the system search is calculated by the following equation.:

(14) $$\begin{array}{c} P=\frac{\text{TP}}{\text{TP}+\text{FP}}. \end{array}$$

Average precision (AP) is the area under the accuracy curve. The higher the AP is, the better the search effect is. The mean average precision (MAP) value represents the average value of AP in multiple categories, and the size of the MAP value is between 0 and 1. The larger the value is, the better the search performance of the system is.

## 3. Result Analysis

### 3.1. Analysis of Experimental Results of Different Datasets

**Figure 8:**
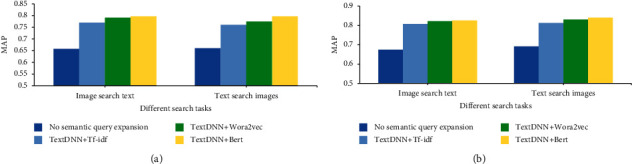
MAP results of search tasks on different datasets. ((a) MAP results for the search task on the dataset of Taylor's University in Malaysia (NUS-WIDE); (b) MAP results of search tasks on the public dataset of early childhood enlightenment education published by the Ministry of Education).

The datasets studied are the public dataset of early childhood enlightenment education published by the Ministry of Education and the dataset of Taylor's University in Malaysia. Different word embedding algorithms are adopted, and 519648 datasets are retrieved. 15000 datasets are extracted from different datasets for training.  Figure 8 shows the search MAP results of different datasets on the DNN-based cross-media education big data intelligent search system.

Figure 8 displays that without semantic query extension, the average mapping of search tasks on the two datasets is between 0.658 and 0.69. Through task search on the system, the MAP performance has been improved by 15.6% on the public dataset of early childhood education published by the Ministry of Education. In addition, the MAP performance of the constructed dataset in the field of early childhood education has also been significantly improved; that is, on the NUS-WIDE dataset, the MAP performance has been improved by 20.6%. To sum up, the word embedding algorithm can improve the search accuracy of depth model task search to a certain extent. However, it is more obvious that the establishment of the system can significantly improve the search accuracy of big data of early childhood education.

### 3.2. Analysis of Intelligent Search Results of Different Algorithms on Different Datasets

**Figure 9:**
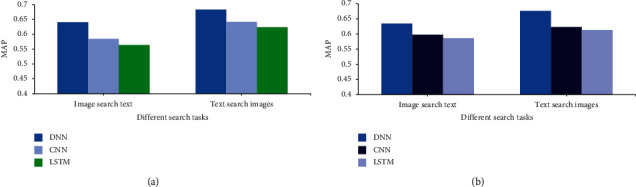
Intelligent search results of different algorithms on different datasets. ((a) MAP results for the search task on the dataset of Taylor's University in Malaysia (NUS-WIDE); (b) MAP results of search tasks on the public dataset of early childhood enlightenment education published by the Ministry of Education).

DNN, CNN, and long short-term memory (LSTM) are used to carry out the intelligent search of children's enlightenment education of cross-media education big data to analyze the comparability of the experimental methods.  Figure 9 shows the intelligent search results of different algorithms on different datasets.

Figure 9displays that the intelligent search results of different neural network algorithms in cross-media education big data for children's enlightenment education are different. Based on datasets (a) and (b), the MAP value of DNN in the search task is obviously the largest, with an average of 0.658, indicating that the search effect is also the best. The second is the MAP value of CNN in the search task, with an average value of 0.611. The average value of the LSTM MAP in the search task is 0.596, with the lowest value.

To sum up, the word embedding algorithm can improve the search accuracy of depth model task search to a certain extent. However, it is obvious that the establishment of the system can significantly improve the search accuracy of big data for early childhood education. In the cross-media big data intelligent search system for early childhood education, DNN has the best search effect. The results show that the research methods proposed are universal and can play an important role in early childhood education.

## 4. Conclusion

This exploration aims to study the value orientation and essence of early childhood enlightenment education based on DNN. Based on the acquisition and feature learning of cross-media education big data and the DNN-related learning of cross-media education big data, a DNN-based cross-media big data intelligent search system for early childhood education is designed and implemented. In addition, the research objects are the public dataset of early childhood education published by the Ministry of Education and the dataset of Taylor's University in Malaysia (NUS-WIDE). Next, the intelligent search results of different datasets in the system and the intelligent search results of different neural network algorithms on cross-media education big data for early childhood education are analyzed. The results show that without semantic query extension, the average MAP of search tasks on the two datasets ranges from 0.658 to 0.69. Through task search on the system, MAP performance is improved by 15.6% on the public dataset of early childhood education published by the Ministry of Education and 20.6% on the dataset of Taylor's University in Malaysia (NUS-WIDE).

This exploration has established a big data intelligent search system for cross-media early childhood education based on DNN. It has certain theoretical significance and empirical value for studying early childhood enlightenment education. However, there are still some deficiencies and limitations in the research process. The main limitation is that there is no empirical research on early childhood education search assisted by different algorithms in the DNN-related learning process of cross-media education big data. Next, for the search results, the purpose of early childhood education has not interacted with it, which may lead to the deviation of users' search intention. In the future, interactive learning can be carried out on the intelligent search of early childhood education to achieve accurate early childhood education.

## Figures and Tables

**Table 1:** Common neural network structure.

Neural network structure	Explanation
Feedforward neural network	The feedback signal is only in the training process. In the classification process, the data can only be transmitted forward to the output layer, and there is no backward feedback signal between layers.
Feedback neural network	The structure is complex, such as the Elman network and Hopfield network.
Ad hoc	No tutor learning. It is self-organizing and adaptive to change the network structure.

## Data Availability

The dataset used in this paper is available from the corresponding author upon request.
